# Crustal Structure of the Collision-Subduction Zone in South of Iran Using Virtual Seismometers

**DOI:** 10.1038/s41598-019-47430-y

**Published:** 2019-07-26

**Authors:** Taghi Shirzad, Mohammad-Ali Riahi, Marcelo S. Assumpção

**Affiliations:** 1 Institute of Astronomy, Geophysics and Atmospheric Science, University of Sao Paulo, Sao Paulo, Brazil; 2 Institute of Geophysics, University of Tehran, 14155–6466 Tehran, Iran; 3 Institute of Geophysics, University of Tehran, 14155–6466 Tehran, Iran; 4 Institute of Astronomy, Geophysics and Atmospheric Science, University of Sao Paulo, 05508-090 Sao Paulo, Brazil

**Keywords:** Seismology, Tectonics

## Abstract

Improving the resolution of seismic tomography by adding virtual seismometers is an ambitious aim in regions with poor instrumental coverage. In this study, inter-event empirical Green’s functions (EGFs) were retrieved using cross-correlation of the vertical component of 630 earthquakes with **M** ≥ 4 which occurred around the collision-subduction transition zone in south Iran. To extract reliable inter-event EGFs and obtain stable tomographic results, we used about 1300 event pairs with good signal-to-noise ratio, each pair well aligned to a seismic station. Our results show that the retrieved inter-event EGFs agree well with those obtained from earthquakes in similar paths. The inverted velocity model presents two main layers including upper crust (up to ~16 km) and middle crust (deeper than ~18 km) in both sides of the Minab-Zendan-Palami transition zone. The upper crust contains two main layers: sedimentary and basement layers with thicknesses ~6 and ~10 km, respectively. Moreover, the main faults cause lateral variations in these main layers. The difference between the average velocities of the middle crust, between the collision and subduction zones, is about 0.5 km/s, delimited by faults. Also, an area with a 30 km width along these faults can be defined as the collision-subduction transition zone.

## Introduction

Seismologists have applied many different techniques (e.g., receiver functions, ambient seismic noise, etc.) using seismic waveforms recorded by real receivers to study the Earth’s interior. Traditional source-receiver observational methods in seismic tomography cannot fully exploit all information contained in a network of sparse stations because of possible ill-posed inversion problems (e.g.^1^). Cells with few or no crossing raypaths lead to artifacts and smearing effects. Poor geometry of earthquakes and stations (non-uniform distribution) can happen near active faults, inside seismotectonic zones in local and/or regional scale tomography, and near plate boundaries in global scale tomography (see^2^ and^3^). Moreover, ambient seismic noise and teleseismic observations may not be sufficient to improve the coverage in poorly resolved cells. However, the resolution of tomography inversion in these regions can be improved using earthquakes as virtual seismometers^4^.

Earthquake event-pair cross-correlation (hereafter ECC) yields a trace similar to real empirical Green’s function (EGF) of the inter-event path, as shown by^5^. Based on source-receiver reciprocity theorem, one of the earthquakes is taken as a source, and the other one is considered a (virtual) receiver recording the waveform of the source. This earthquake interferometry approach provides inter-event EGF that is useful to study the Earth’s crustal structure where it is impossible to directly record waveforms, such as in fault zone tomography (see^6^ and^7^).

**Figure 1:**
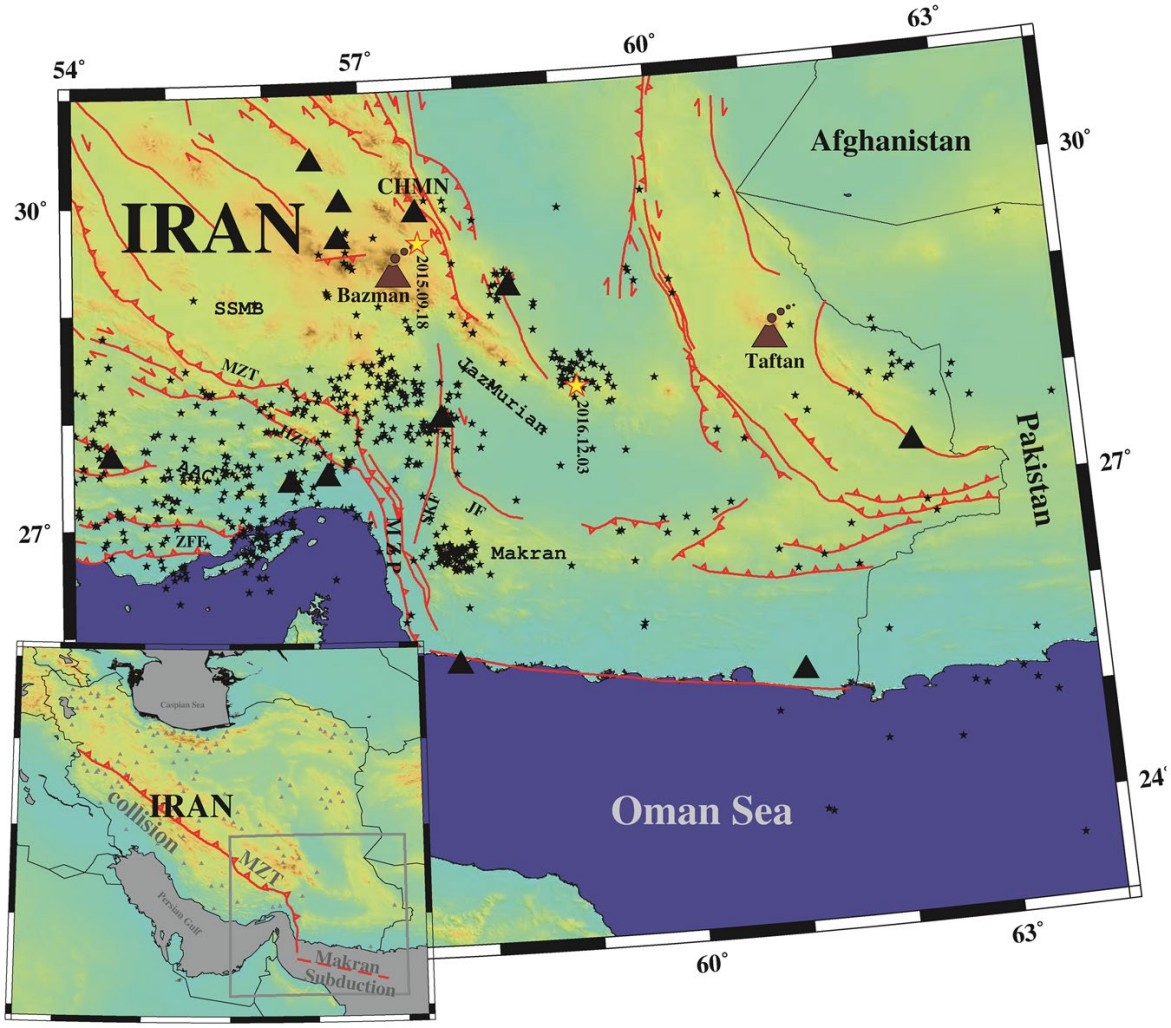
Map of the study area showing the main geographical and tectonic features mentioned in the text. Stations, earthquakes epicenters and active faults depicted by black triangles, stars, and solid red lines, respectively. Abbreviations are AAC = Afro-Arabian Continent, SSMB = Sanandaj-Sirjan-Metamorphic Belt, MZT = Main Zagros Thrust, HZF = High Zagros Fault, MZP = Minab-Zandan-Palami fault, SKF = Sabzevaran-Kahnuj-Fault, JF = Jiroft Fault, Jazmurian = Jazmurian depression, and ZFF = Zagros Foreland Folded. Active Bazman and Taftan stratovolcanoes show by brown color. The bottom left map gives the location of the study area. Moreover, in this inset figure, abbreviations MZT is Main Zagros Thrust.

Convergence between the Arabian and Eurasian plates controls the main tectonic activity characterized by continent-continent collision in western Iran (along the Zagros Collision) and subduction in southern Iran along the Makran subduction (Fig. 1, inset map). A GPS-measured convergence rate varies from ~10 mm/y across the Main Zagros Thrust in Iran, part of the collisional system (with NNE shortening) to ~28 mm/y in the Makran subduction^8^. The transition zone between the Zagros collision and Makran subduction is known as Minab-Zendan-Palami (hereafter MZP) fault zone with ~25 mm/y (see^9^ and^10^). This reverse and right-lateral, NNW trending fault system, offsets the topographical deformation in the Zagros and the Makran prisms^11^. Many studies have been made on this transition zone, such as stress distribution (see^10,12^ and^13^), source parameters and/or slip distribution (e.g.^14^), joint gravity and surface wave tomography^15^, and ambient seismic noise tomography (e.g.^16^ and^17^). However, few results about crustal structure have been published. Also, most of the regional tomographic results (e.g.^18,19^ and^20^) are limited due to low spatial sampling in the crust and broad lateral resolution.

In the following sections, we extract inter-event EGF waveforms and measure their dispersion curves to construct Rayleigh wave tomographic images in south Iran. Our results indicate that the MZP fault zone generally separates two distinct crustal blocks with different velocity anomalies in each side of the fault line.

## Dataset and Data Selection

We used the vertical component (Z) of mainshock waveforms with Nuttli magnitude (see^21^), **M**_N_ ≥ 4.0 recorded by Iranian Seismological Center (IrSC) network. The stations were equipped with short period (SS-1 Kinemetrics) and broad band sensors and the earthquakes were recorded from 2006 January to 2019 May. All waveforms were decimated to 10 sps. Figure 1 shows the stations and earthquakes (triangles and stars, respectively).

We selected well-recorded earthquakes with waveforms taken from the origin time to the end of the Rayleigh wave coda. Waveform with total time gaps (Δ_t_) larger than 5 s were rejected to avoid artifact/underestimated anomalies at shorter periods. Notably, non-recording of data by the station in a few seconds leads to this type of gaps. In addition, we selected good quality locations with *rms* of time residuals less than 0.2 s (termed RMS_location_ < 0.2 s^22^), epicentral uncertainties less than 8 km, recorded by at least ten stations. As the fourth selection criteria, we used waveforms with the epicentral distances greater than 15 km (Δ ≥ 15). The final selection criterion, before signal preparation, was the waveform signal-to-noise ratio, which should be greater than 4 (SNR_event_ ≥ 4). In this study, the SNR value is the ratio of the peak of the signal envelope (from the P arrival to the time corresponding to velocity 2.0 km/s) to the root-mean-square of the noise window (1.3 to 1.6 km/s), filtered in period range of 4 to 26 s^23^.

## Method

### Retrieving inter-event EGF

**Figure 2:**
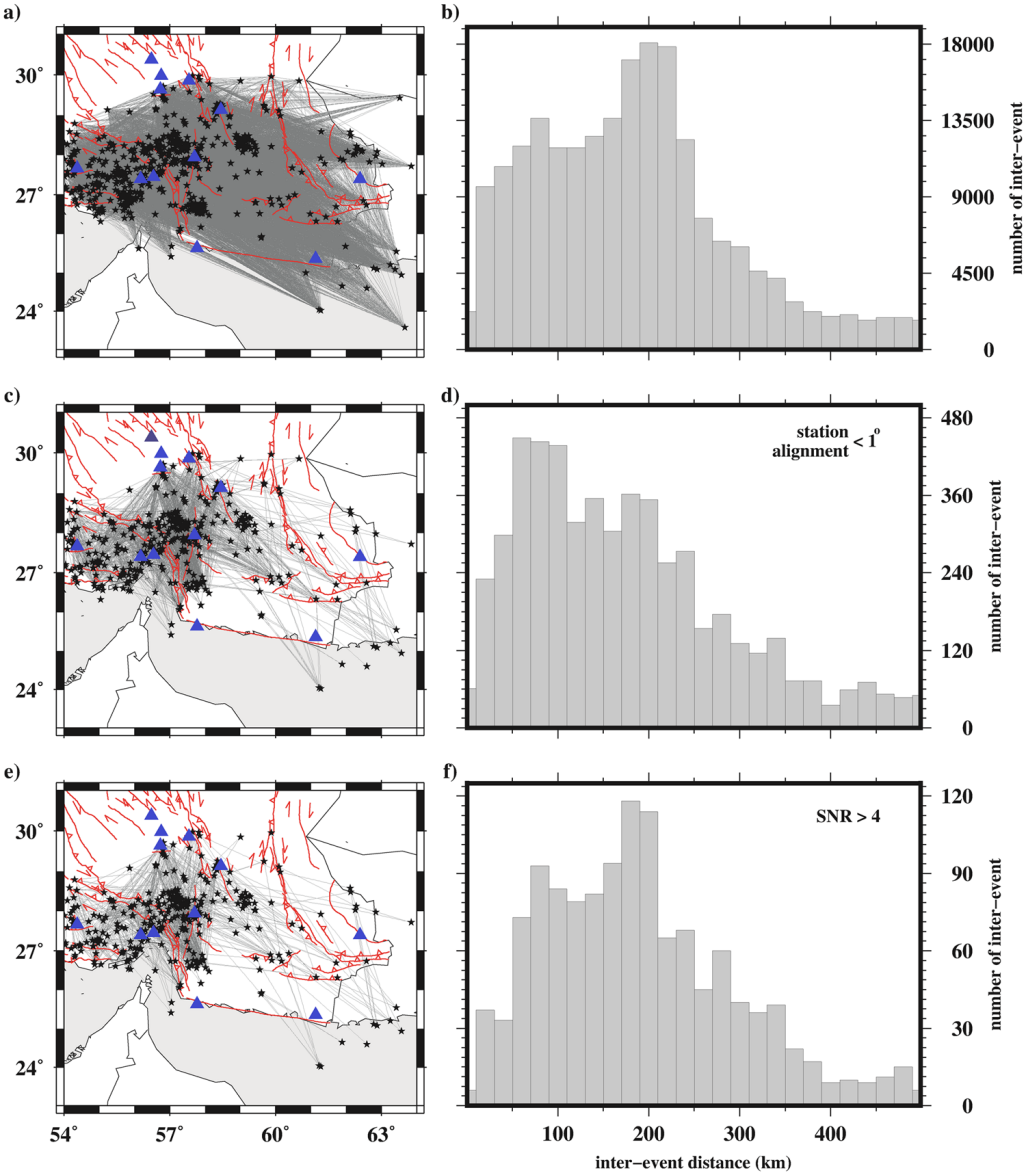
All possible paths for earthquake-pairs is shown in (**a**), and the histogram of their inter-event distances in (**b**). All event-pair raypaths, aligned with a seismic station within 1° are depicted in (**c**) and the corresponding histogram for inter-event distances in (**d**). This constraint leads to decrease the ray coverage from 234,978 (**a**) to 8,091 (**c**) paths. All signals in (**c**) with SNR less than four are rejected to obtain stable tomographic maps that the remaining raypath coverage shows in (**e**), and a histogram of corresponding distances in (**f**). Cross-correlating, stacking and dispersion measurements were computed for these 1,259 signals in the inter-event distance range of 50 to 350 km. Some event pairs are aligned with a seismic station outside the plotted area (see inset of Fig. 1 for all Iranian stations).

The EGF (in earthquake, and ambient seismic noise studies) contains mainly the fundamental mode Rayleigh wave because of its large amplitude, and surface wave traveltime tomography are routinely used to determine velocity structure from local scale (e.g., mostly upper crust by^24^) to continental scale (e.g., crustal structure by^25^ and^26^). The basic assumption of the earthquake interferometry is that the ECC from a pair of the events recorded at the same receiver is retrieved as a waveform signal that is propagated between the earthquake event-pair^5^. All available ECC (after the selection criteria described above) were calculated as depicted by the ray paths in Fig. 2a. To reduce the contribution of the non-stationary energetic arrivals, we only use stations which deviate less than 1° from the event-pair alignment, as shown in Fig. 2c. Although using all available event-pairs (see Fig. 2d) could provide a higher lateral resolution, we only used event-pairs with distances (Δ) constrained by maximum wavelengths greater, λ_max_, = Δ/3 (as recommended by^27^).

**Figure 3:**
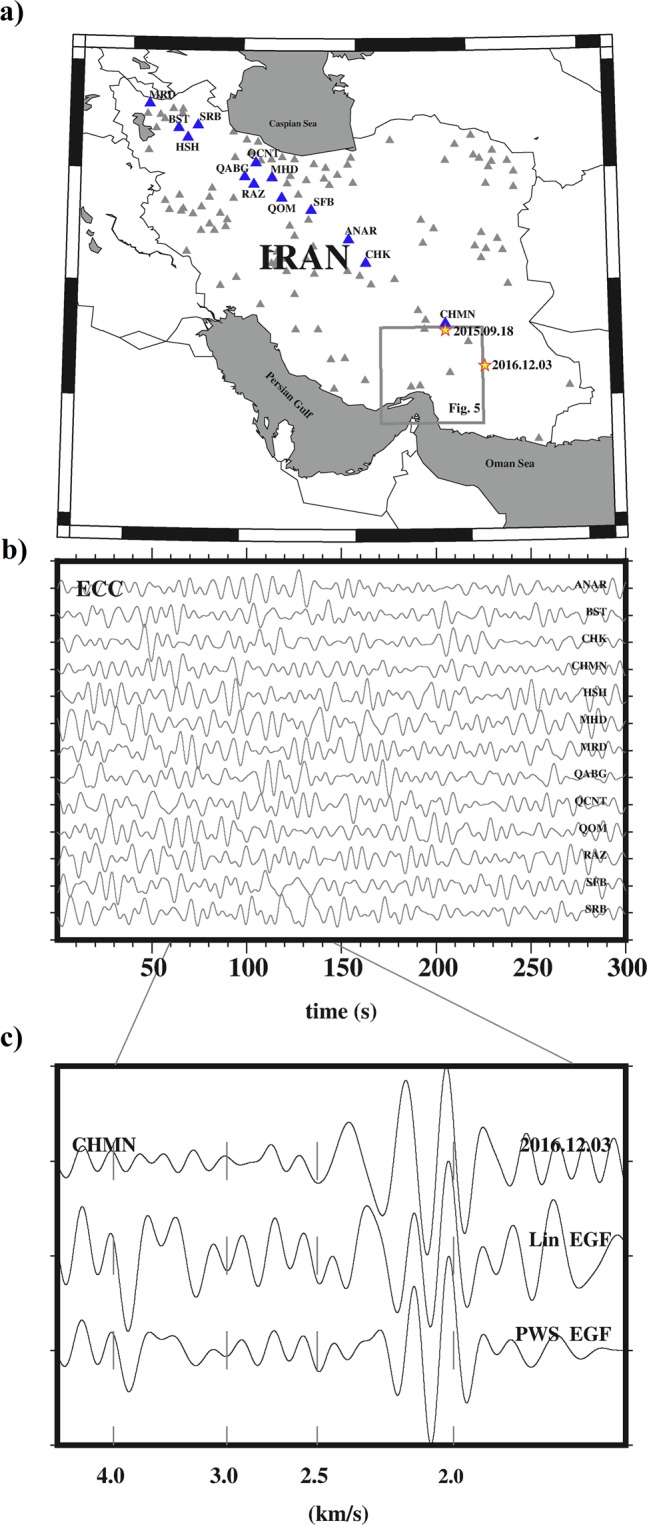
(**a**) Stations of the IrSC network with the two earthquake pair shown as red stars. (**b**) cross-correlation (ECC) traces in the period range of 4–26 s for all available stations in the stationary zone, i.e., the station labeled in part (**a**). (**c**) Comparison of linear (LIN) and phase weighted (PWS) stacking of ECC with Rayleigh wave excited by an earthquake (2016.12.03; referred in Fig. 1). All traces were normalized to the maximum amplitude of PWS stacking signals.

Figure 3 shows an example of cross-correlation between a pair of earthquakes in southern Iran. Because surface waves dominate the seismograms, the surface wave part of inter-event signals is the first to emerge from the ECC^28^, if all stations are along the event-pair raypath (inside stationary zone). As an example, we used 13 stations (blue triangles in Fig. 3a) aligned with two earthquakes which occurred on 2015.09.18 and 2016.12.03 (red stars in Fig. 3). It is assumed that the records do not include multi-pathing from source to receivers (^29^ and^30^). The earthquake waveforms (for each stationary station) are firstly prepared using the common low-frequency technique outlined in^28^. Therefore, all time gaps (Δ_t_ ≤ 5 s) were filled with zero, mean and trend were removed, and bandpass filter was applied in the period range 4 s to 26 s. To amplify the contribution of the low/weak energetic arrivals and to homogenize the effect of periods with high energy, we applied time and frequency domain normalization (^29^ and^31^). Finally, the prepared signals were cross-correlated to obtain the ECCs. All available ECCs for the 2015.09.18–2016.12.03 pair corresponding to stations (see Fig. 3a) aligned in the stationary zone are shown in Fig. 3b.

Afterwards, the calculated ECC signals are stacked by linear (hereafter LIN^32^) and phase weighted (hereafter PWS^33^ and^34^) stacking methods. The stacked ECCs of LIN and PWS are compared to the earthquake record (event on 2016.12.03 recorded by CHMN station) in Fig. 3c. The Rayleigh wave observed in the inter-event EGF (LIN and PWS) is similar to that of the real earthquake with about the same ray path. By repeating these processes, we computed all available event-pair EGFs depicted in Fig. 2e.

### Dispersion measurement

The phases of the LIN and PWS signals are not completely similar to the real earthquake trace. This difference may be caused by the slightly different source-receiver distances or discrepancy between obtaining strain and displacement as mentioned by^5^. But, the significant similarity between the amplitude (energy) part of virtual and real EGFs is clear, so that the energy part of virtual EGFs are reliable and characterize the inter-event structure. Therefore, we used the Rayleigh wave group velocity rather than phase velocity dispersion measurements for further processing. Moreover, although the inter-event EGFs extracted by both the LIN and PWS methods contain the same Rayleigh wave signal, the PWS stack has higher SNR, and was used for the dispersion measurements.

**Figure 4:**
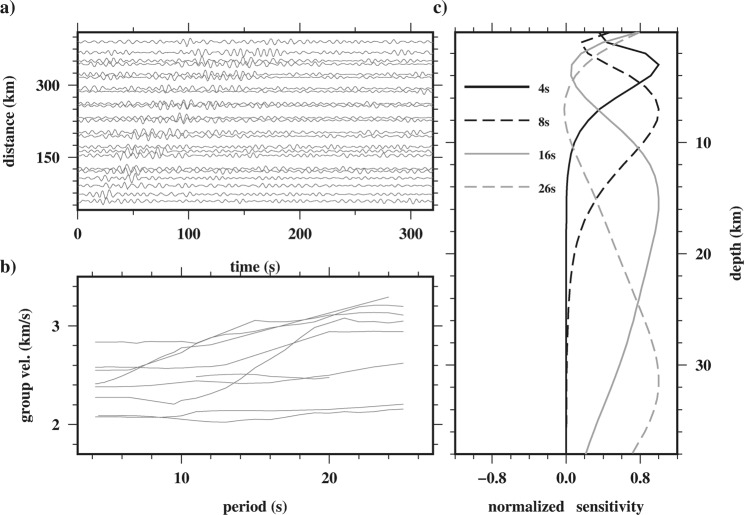
(**a**) Selected (a signal every 25 km) inter-event EGF signals. (**b**) Examples of dispersion curves used in the tomography. (**c**) Normalized sensitivity kernel as a function of depth at the periods of 4, 8, 16 and 24 s.

We only used inter-event EGFs with SNR higher than 4 (SNR_EGF_ ≥ 4), and inter-event distance larger than three wavelengths (Δ ≥ 3λ) to measure the group velocity dispersion^35^. Thus, 1,259 inter-event raypath (Fig. 2e) from 630 well-constrained earthquakes were used for the tomography. This constraint reduces the raypath coverage as compared in Fig. 2d,f but it is necessary to retrieve reliable inter-event EGFs (^35^ and^36^) and to stabilize the tomography. Figure 4a shows a record section of selected inter-event EGF (one signal every 25 km).

To obtain Rayleigh wave group velocity, U, dispersion measurement, 1,259 waveforms were manually picked by Computer Program in Seismology^37^. Because this program uses a set of Gaussian filters and phase matched filter (^38^ and^39^), the best trade-off between resolution in frequency and time domains is obtained by selecting a suitable Gaussian filter, α, which depends on the inter-event distance^40^. Therefore, α was set to 3 for the inter-event distances less than 200 km and a value of 6 for larger distances^41^. Some selected Rayleigh wave group velocity dispersion curves are shown in Fig. 4b.

### Sensitivity kernel

Different periods of surface wave sample different depth ranges (see^42^). In other words, short period dispersion is sensitive to upper crustal structure, while longer periods are needed to sample greater depths. Thus, the sensitivity kernel for each period as a function of depth defines the maximum penetration depth. Figure 4c shows the sensitivity kernel as a function of depth for periods of 4 (solid black), 8 (dashed black), 16 (solid gray) and 26 (dashed gray) s. Although minimum and maximum penetration depths are approximately 1 and 40 km, the sensitivity of the fundamental mode Rayleigh wave for each period is negligible around the maximum penetration depth. Therefore, the effective depth range of the sensitivity kernels was used for further processing and interpretation in this study, which is in the range of ~2 to 30 km.

### 2D tomography

**Figure 5:**
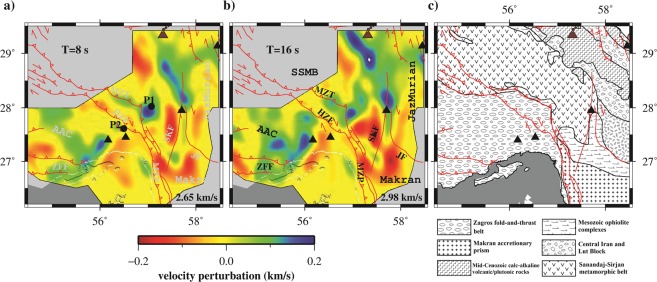
Group velocity maps obtained by event interferometry approach in the periods of (**a**) 8 and (**b**) 16 s. Red solid lines and black triangles show known active faults, and stations used in the study area, respectively. The background velocities are 2.65 and 2.98 km/s for 8 and 16 s, respectively. (**c**) Main geological units.

In this study, the dispersion measurements were inverted to obtain group velocity maps using the Fast Marching Surface-wave Tomography method of^43^. The study area was parameterized with 0.2° × 0.2° grid cells, and average value of group velocity, U _average_, used as a initial model for each period. However, to compensate for sparse, non-crossing and different number of ray paths within each grid cell, regularization parameters (e.g., damping and smoothing) were applied. The final optimum regularization parameters were chosen using standard L-curve analysis^44^. These optimal parameters increase the robustness of the inversion procedure and result in stable tomographic maps. Figure 5 shows the Rayleigh wave tomographic map for periods of 8 and 16 s. The variance reductions of the tomographic inversion were 73% and 78% at 8 and 16 s, respectively.

**Figure 6:**
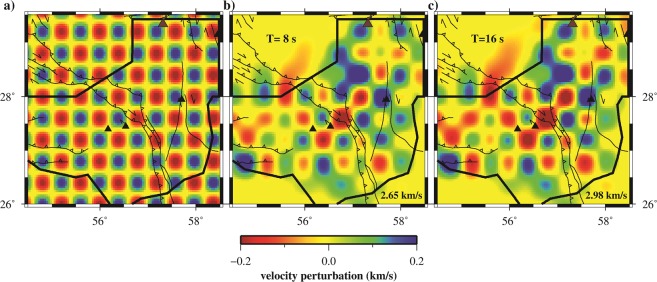
(**a**) The initial model for checkerboard resolution test of group velocity. Recovered results for the periods of (**b**) 8 and (**c**) 16 s. Stations and active faults depicted by black triangles and lines, respectively. The black thick solid border separates area with insufficient lateral resolution. The background velocities are 2.65 and 2.98 km/s for 8 and 16 s, respectively.

As shown in Fig. 5a which is sensitive to a depth of ~7 km, (see Fig. 4c) a low-velocity basement is observed in the Sanandaj-Sirjan metamorphic belt (SSMB; around 56.8 E and 29.0 N), Makran accretionary prism, and Jazmurian depression. While the Zagros Fold and Thrust Belt (hereafter ZFTB, the area south of the MZT), some part of the Mesozoic Ophiolite complexes and south part of Lut block (around 57.8E and 28.0 N) show high-velocity anomalies. Moreover, Fig. 5b (sensitive to the middle crust, see Fig. 4c), contains low velocity structures in the SSMB and some part of NW Makran (subduction zone) which may imply a thinner upper crust. Also, high velocity structure clearly appeared in the ZFTB where a thick upper crust may exist. However, the geometry of the inter-event raypaths affects the lateral resolution of tomographic results. To test the robustness of the inversion, we carried out a checkerboard resolution test with similar grid size (Fig. 6a) and the same regularization parameters (e.g., damping and smoothing) of tomographic maps. The recovered resolution maps indicate that the regularization parameters prevent resolution of sharp discontinuities at the NW, SW and SE corners of the study area. The area with reasonable resolution in Fig. 6b,c is limited by the thick solid black line, and the area outside this polygon was masked in Fig. 5.

### Shear wave velocity

**Figure 7:**
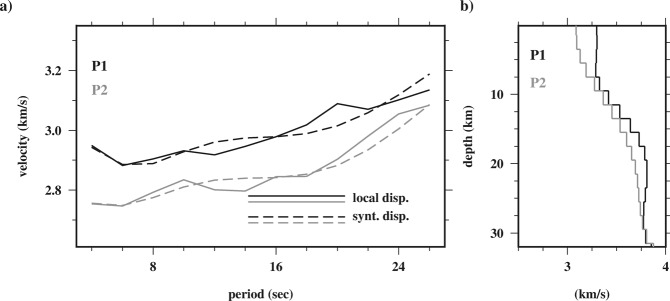
(**a**) Pure group velocity dispersion calculated at geographic grid cell **P**1 (57°E and 28°N; referred in Fig. 5a) and **P2** (56.5°E and 27.6°N; referred in Fig. 5a). (**b**) 1D shear velocity profile at **P1** (black) and **P2** (gray). The synthetic predicted dispersion curves are depicted by dashed lines in (**a**).

**Figure 8:**
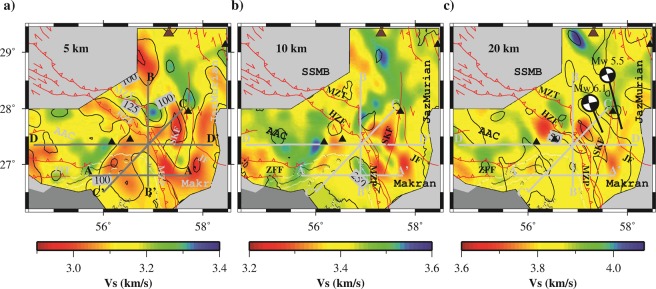
(**a**) Horizontal slices of shear wave velocity at depths of 5, 10 and 20 km. Two focal mechanisms of earthquakes occurred on 2013 May 11 at 02:08:08 (**M**_W_ 6.1) and 2013 May 11 at 03:09:47 (**M**_W_ 5.5) corresponding to SKF and JF, respectively. Black contours represent the estimated initial model uncertainties.

To obtain a crustal shear wave velocity model, the Rayleigh wave group dispersions were constructed for each grid cell (0.2° × 0.2°) in the period ranges of interest, as described by^45^. For instance, Fig. 7a shows the dispersion curves at grid cell **P**1 (57°E and 28°N) and **P2** (56.5°E and 27.6°N) referred to in Fig. 5a. Each dispersion curve was inverted using an iterative damped least-squares inversion procedure^37^ to determine the best fitting V_S_ model. During the 1D V_S_ inversion, the initial model was parameterized with multiple layers (constant thickness = 1 km) over a half space, with fixed V_P_/V_S_ ratio (≈1.7), and density using the relation in^46^. Figure 7b shows an example of 1D V_S_ model at grid cells **P1** (black line) and **P2** (gray line). Finally, by repeating this process for all grid cells a quasi 3D V_S_ model was constructed. Figure 8 shows the maps of V_S_ at depths of 5, 10 and 20 km.

## Results

We present examples of the shear wave velocity profiles in the region with credible resolution. The qualitative discussion of the structural features that appear in these profiles and tomographic maps will be also presented in the next section.

**Figure 9:**
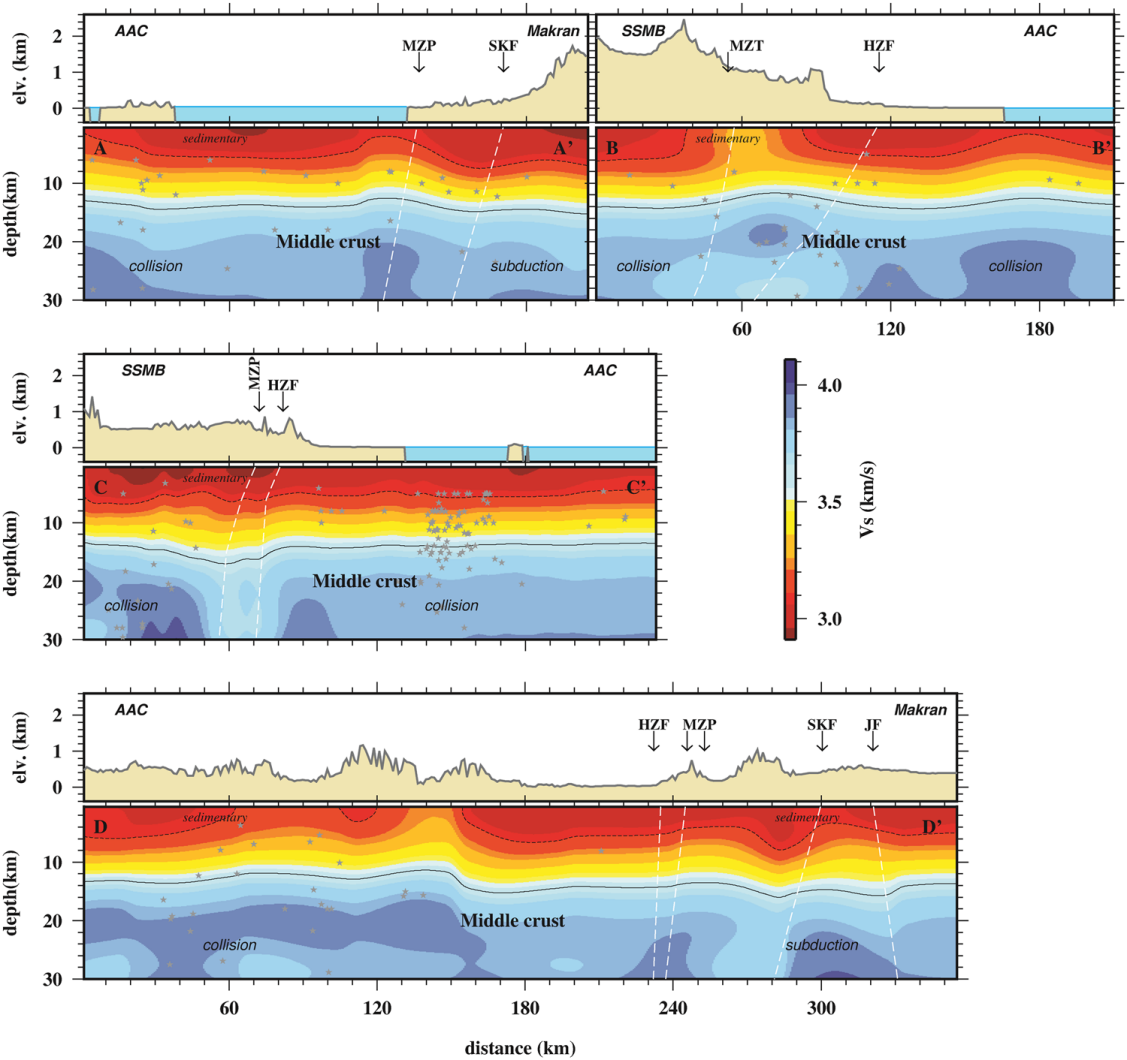
Vertical cross-section of shear wave velocity for four transects referred to in Fig. 8. Gray stars indicate earthquake hypocenters, and following up known faults from the surface to depth using edges of clutter velocity layers are represented by dashed lines.

Figure 9 shows four vertical V_S_ profiles across and along the transition between continent-collision and subduction zones which indicate a low-velocity anomaly (<3.1 km/s) confined within the upper 6 km. The thickness of the low-velocity layer varies up to 6 km and corresponds to the thick sedimentary layer as observed by^15^. This sedimentary layer overlies a basement layer with many structural features of deformation, fold, and fracture. As shown in these profiles, most earthquakes, M ≥ 4.5 (gray stars), occur in this basement layer. In brief, the upper crust consists of two distinct layers including a sedimentary layer (without any earthquake **M** ≥ 4.5; see gray stars in Fig. 9) and basement layer. These profiles were selected to explain the tectonic features in areas with good/fair resolution based on the checkerboard test (see Fig. 6).

The E-W profile AA’ (along 55.7°E, 26.8°N to 57.7°E, 26.8°N; referred to in Fig. 8) shows that the sea side of collision zone beneath the Afro-Arabian continent (hereafter AAC) separates by nearly vertical high-velocity anomaly zone (in distances ~120 km beneath MZP) from Makran subduction. Although the Sabzevaran-Kahnuj Fault (in distance of 170 km; hereafter SKF; at thick sedimentary beneath Makran) does not change the velocity layering in upper crust (especially sedimentary layer), it leads to the separation of velocity structure in middle crust within the subduction zone. The N-S profile BB’ (along 56.9°E, 28.5°N to 56.90°E, 26.6°N; referred to in Fig. 8) start in the SE part of SSMB (on the side of Iranian micro-continent), cuts MZP and High Zagros Fault (hereafter HZF), and across through collision zone before reaching the eastern edge of AAC. The Main Zagros Thrust (hereafter MZT) clearly appears as a relatively high-velocity zone between SSMB and AAC in the distance range of 40 to 80 km.

The third profile is CC’ (from 57.6°E, 28.0°N to 56.0°E, 26.5°N; referred in Fig. 8) which is running along trending convergence direction from SW to NE crossing collision zone. In this profile, high deformation in velocity layers appears in the basement of the upper crustal layer. Whereas, the middle crust contains approximately uniform velocity structure in the two sides of the collision zone, except around the MZP and HZF. The final profile is the DD’ (from 54.6°E, 27.35°N to 58.2°E, 27.35°N; referred in Fig. 8), ~350 km long, which crosses the land side of continent collision and subduction zones. Regardless of length comparison of profiles AA’ and DD,’ the sedimentary layers (V_S_ ≤ ~3 km/s) vary between 4 to 6 km from collision side to subduction zone. As shown in these profiles, the collision-subduction transition zone is varied along the MZP with a width range of 20 to 40 km.

## Discussion

We used earthquake interferometry approach to recover crustal structure around the MZP fault zone where the Makran subduction is separated from the Zagros continent collision zone. Non-stationary waveforms are one of the main difficulties with the event-pair interferometry. Using stations along inter-event raypath, strict event selection criteria (e.g., SNR_event_ ≥ 4, location uncertainty < 8 km, RMS_location_ < 0.2 s), and tomographic criteria (e.g., Δ ≥ 3λ, SNR_EGF_ ≥ 4, RMS_tomography_ residual < 15 s, and U(T) ≤ U _average_ (T) ± 2σ(T), where T is the period and σ is standard deviation of U) ensured the quality and stability of tomography inversion results. Moreover, to consider the reproducibility, our analysis indicated that the standard deviations of the dispersion measurements of each ECC in Fig. 2b were almost in the range of velocity measurement error bars.

High correlations with the major tectonic and geological features are recognizable in the shear wave velocity result at a depth of 5 km (Fig. 8a). Low-velocity anomalies appear in the SSMB and Makran zone which are caused by crustal thickening inner collision zone^16^ and thick sedimentary in forearc basin (~6 km thickness^47^ and^48^), respectively, whereas, high-velocity anomalies emerge in the ZFTB (in the continent collision part^17^) and southern part of Bazman volcano (in volcano arc^49^). These results agree well with joint inversion of gravity-surface wave tomography results^15^. However, for greater depths (Fig. 8b,c), relatively low-velocity anomaly in SSMB could be interpreted as a duplex structure between MZT and HZF which was suggested by^50^. Moreover, the main tectonic features, e.g., AAC, SSMB, Makran, Jazmurian depression, or Lut block edges (around 57.8E and 28.0N) appear as boundaries of high or low-velocity anomalies boundaries. The duplex system branches from a single fault in the depth and merges imbricate (overlapping) thrusts in the subsurface. To assess the influence of the V_S_ initial model, a bootstrapping stochastic test was done. This parametric test guarantees the reliability of the calculated V_S_ model^51^. For this purpose, a normal random distribution with a standard deviation of 0.3 km/s was applied to obtain 300 different initial V_S_ models. Iterative damped least-square inversion procedure was then inverted each local group velocity dispersion curves by these perturbed initial models. Finally, standard deviations of the inverted shear wave velocity were calculated at each grid point. Black counters in Fig. 8 represent the spatial distribution of these standard deviations. As shown in this figure, these uncertainties vary between 50 (see Fig. 8a,c) and 250 (see Fig. 8b) m/s.

Inspection of AA’ (Fig. 9) indicate that the SKF does not have a clear surface expression in the southern part, so that some studies (e.g.^14^) related some earthquakes around it to the left lateral strike-slip such as 2013 May 11 (M_W_ 6.1 at a depth of 20 km). As shown in profile BB’ (Fig. 9), the event location and low velocity (especially in the middle crust) could be signatures of a duplex system between MZP and HZF which separates two shields as explained by^50^. Also, the higher velocity appears in middle crust beneath the AAC which is in agreement with previous studies (e.g.^18,19^ and^20^). The strongest low-velocity anomaly in the sedimentary layer is also seen beneath the SSMB (on the side of Iranian micro-continent) than the AAC. Whereas, in profile CC’ (Fig. 9), crustal thickening, folding and deformation in the foreland (AAC) may cause more seismicity (gray stars) than the hinterland (SSMB). In this profile, the irregular/disordered velocity layers up to 6 km are the main characters in the sedimentary layer beneath SSMB (distance range of 20 to 100 km) which is consistent by duplex theory in the Zagros Mountains (see^50^). The profile DD’ (Fig. 9) clearly shows that the mountain belt (distance range of 100 to 200 km) in the collision zone, and HZF, MZP, SKF and JF in the subduction zone cause disorder in the sequence of the upper crust layers. Also, in the middle crust of the collision zone, shear wave velocity is slightly greater than the subduction side. Moreover, the dip angles of SKF and JF are opposing which agrees with the earthquake focal mechanisms on these faults. As reported by USGS, these earthquakes occurred on 2013 May 11 at 02:08:08 (**M**_W_ 6.1; see Fig. 8c) and 2013 May 11 at 03:09:47 (**M**_W_ 5.5; see Fig. 8c) corresponding to SKF and JF, respectively^52^.

## Conclusion

The use of virtual seismometers, or event interferometry approach, covers several shortcomings of traditional earthquake-receiver and ambient seismic noise tomography methods. The results of this study can be summarized as below:

1. This method can produce high-resolution tomographic maps in the seismic region with sparse stations but many events. Therefore, short period surface wave group velocity maps can be obtained by this method which, otherwise, are very difficult to obtain with traditional/classical tomography (e.g., source-receiver, ambient seismic noise, teleseismic and so on).
2. Reliable inter-event EGFs were retrieved by stations located along the event-pair directions using strict constraints on recorded waveforms and on cross-correlated signals (see Fig. 3).
3. The group velocity tomographic pattern agrees with the main geological units as shown in Fig. 5. The shear wave velocity model is consistent with previous results (e.g.^15^), and also with the known main tectonic features (see Figs 8 and 9). The V_S_ profiles agree well with the expected thickness of the sediments in the collision and subduction zones. These sedimentary layers clutter/interfuse around main faults (e.g., MZP). The V_S_ profiles showed that the shear wave velocity in the collision zone is slightly greater than the subduction zone. In general, the tomographic maps and profiles agree well with the tectonic features and seismicity of the study area, and provide new information about the collision-subduction transition zone.
4. Using earthquakes as virtual sources shows great potential to be used in other areas with many earthquakes and sparse stations.

## Data Availability

The digital earthquake dataset is available to the public in the Iranian Seismological Center (IrSC) at the University of Tehran/Iran (http://irsc.ut.ac.ir).
